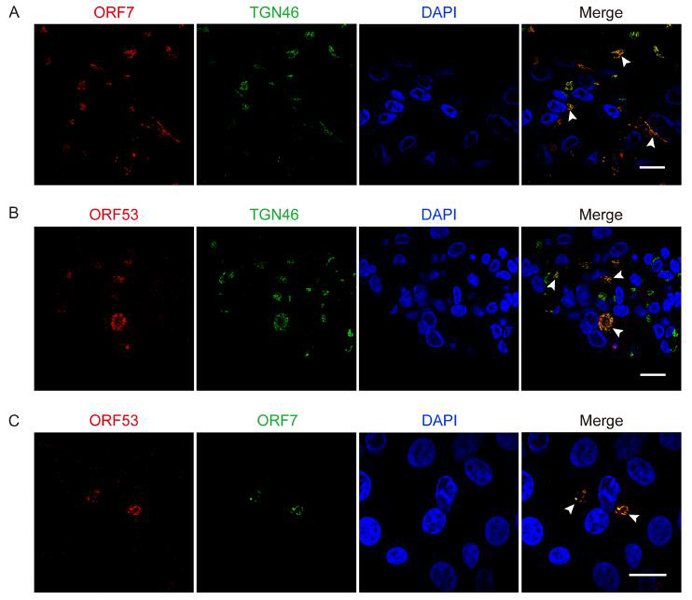# Varicella-zoster virus ORF7 interacts with ORF53 and plays a role in its trans-Golgi network localization

**DOI:** 10.1007/s12250-017-4048-x

**Published:** 2017-10-30

**Authors:** Wei Wang, Wenkun Fu, Dequan Pan, Linli Cai, Jianghui Ye, Jian Liu, Che Liu, Yuqiong Que, Ningshao Xia, Hua Zhu, Tong Cheng

**Affiliations:** 1 State Key Laboratory of Molecular Vaccinology and Molecular Diagnostics, National Institute of Diagnostics and Vaccine Development in Infectious Diseases, School of Public Health, Xiamen University, Xiamen, 361102 China; 2 Department of Microbiology, Biochemistry and Molecular Genetics, New Jersey Medical School, Rutgers University, Newark, 070101 USA

**Keywords:** varicella-zoster virus (VZV), ORF7, ORF53, protein-protein interaction, trans-Golgi network

## Abstract

Varicella-zoster virus (VZV) is a neurotropic alphaherpesvirus that causes chickenpox and shingles. ORF7 is an important virulence determinant of VZV in both human skin and nerve tissues, however, its specific function and involved molecular mechanism in VZV pathogenesis remain largely elusive. Previous yeast two-hybrid studies on intraviral protein-protein interaction network in herpesviruses have revealed that VZV ORF7 may interact with ORF53, which is a virtually unstudied but essential viral protein. The aim of this study is to identify and characterize VZV ORF53, and to investigate its relationship with ORF7. For this purpose, we prepared monoclonal antibodies against ORF53 and, for the first time, characterized it as a ~40 kDa viral protein predominantly localizing to the trans-Golgi network of the infected host cell. Next, we further confirmed the interaction between ORF7 and ORF53 by co-immunoprecipitation and co-localization studies in both plasmid-transfected and VZV-infected cells. Moreover, interestingly, we found that ORF53 lost its trans-Golgi network localization and became dispersed in the cytoplasm of host cells infected with an ORF7-deleted recombinant VZV, and thus ORF7 seems to play a role in normal subcellular localization of ORF53. Collectively, these results suggested that ORF7 and ORF53 may function as a complex during infection, which may be implicated in VZV pathogenesis.

## INTRODUCTION

Varicella-zoster virus (VZV) is a ubiquitous human-specific alphaherpesvirus that causes chickenpox (varicella) and establishes latency in cranial nerve and dorsal root ganglia during primary infection; many years later, this virus may reactivate to produce shingles (herpes zoster) and postherpetic neuralgia (PHN), which is a painful, refractory disease seriously affecting patient's quality of life (Gilden et al., 2000; Cohen et al., 2007; Drolet et al., 2010; Johnson et al., 2010; Weinke et al., 2010; Lukas et al., 2012). In the course of infection, VZV induces pathological abnormalities mainly in skin and nerves. To date, VZV protein function in cells and tissues has been studied extensively by mutagenesis of the VZV genome together with the use of human fetal tissues either cultured *ex vivo* or engrafted in severe combined immunodeficient (SCID) mice *in vivo*, and many viral proteins have been identified to be implicated in VZV skin- and/or neuro-tropism (Zhang et al., 2010; Zerboni et al., 2014; Wang et al., 2015).

ORF7 is a VZV tegument protein that is conserved among all alphaherpesviruses (Jiang et al., 2017). Like its homologue, UL51 of herpes simplex virus type 1 (HSV-1), ORF7 is dispensable for VZV growth in cell culture; however, deletion of ORF7 impairs VZV virulence in *ex vivo* human skin, and thus ORF7 is revealed as one of skin-tropic factors of VZV (Zhang et al., 2010). Moreover, ORF7 deletion severely impairs VZV growth in differentiated SH-SY5Y human neuroblastoma cells and human embryonic stem cell (hESC)-derived neurons *in vitro*, as well as in human dorsal root ganglia (DRG) *ex vivo* and *in vivo*, suggesting that ORF7 is also one of the factors determining VZV neuro-tropism (Selariu et al., 2012). Overall, ORF7 has a critical role in the pathogenesis of both skin and nerve infection of VZV, however, the underlying molecular mechanism have yet to be elucidated.

Identifying and characterizing viral proteins interacting with ORF7 may provide useful information for understanding its biological function at the molecular level. Previous yeast two-hybrid analysis of intraviral protein interactions in VZV has revealed that ORF7 can interact with itself, ORF23 and ORF53 (Uetz et al., 2006; Stellberger et al., 2010), but these interactions have not been further confirmed in the context of VZV infection. In the present study, we focused on the properties of ORF53, which is predicted to be a tegument protein consisting of 331 amino acids and is essential for VZV replication (Zhang et al., 2010), and we explored its relationship with ORF7 in both plasmid-transfected and VZV-infected cells.

## MATERIALS AND METHODS

### Cells and viruses

SP2/0 cells were cultured in RPMI-1640 medium, 293T and ARPE-19 cells were cultured in Dulbecco's modified Eagle medium (DMEM). All media were supplemented with 10% fetal bovine serum (FBS), 50 U/mL penicillin, and 50 μg/mL streptomycin (All from Gibco, Grand Island, NY, USA).

The wild-type parental Oka strain (pOka) was used to generate VZV BAC as previously published (Zhang et al., 2007), but the BAC vector used in this study didn't contain a green fluorescent protein (GFP) expression cassette. Briefly, VZV BAC was maintained and propagated in *E. coli* strain DY380. VZV BAC deleted for ORF7 was created by electroporating PCR amplified kanamycin resistance (kanR) cassettes with homology arms to ORF7 into the VZV-BAC-containing DY380. The correct colonies were selected on kanamycin LB plates and stored in sterile 10% glycerol at –80 °C. Revertant virus was made by rescuing the full-length ORF7 back into the ORF7-deleted VZV BAC. BAC DNA was isolated from *E. coli* and transfected into ARPE-19 cells to generate viruses, including recombinant pOka virus (WT), ORF7 deletion mutant (7D) and its rescue virus (7R). All the viruses didn't express GFP which were different from those reported in our recent work (Selariu et al., 2012; Jiang et al., 2017). VZV were propagated in ARPE-19 cells and stored in liquid nitrogen as cell-associated viral stocks. Titers were determined by infectious focus assay in ARPE-19 cells before use.

### ORF7 and ORF53 expression plasmid construction

The full length genes of ORF7 and ORF53 were amplified by PCR from the extracted genomic DNA of the recombinant pOka virus (WT) or from the isolated VZV BAC DNA. Prokaryotic expression plasmid construction for ORF53 used the forward primer 5′-CGCGGATCCATGCAGCGGATTCGACCTTA-3′ and the reverse primer 5′-CCCAAGCTTTTACTTTACAACCCGTGGTG-3′; *Bam*H I and *Hin*d III restriction sites are underlined. The PCR products were digested with *Bam*H I and *Hin*d III, and then cloned into a pTO-T7 expression plasmid which was previously constructed in our lab (Luo et al., 2000). Eukaryotic expression plasmid for ORF7 and ORF53 was constructed using the following primer pairs, respectively. The forward primer of ORF7 was 5′-TCTAGAGAATTCGGATCCATGCAGACGGTGTGTGCC-3′ and its reverse primer was 5′-CCATGGCTCGAGCCCGGGTTATACAAGCATAACATG-3′; The forward primer of ORF53 was 5′-TCTAGAGAATTCGGATCCATGCAGCGGATTCGACCT-3′ and its reverse primer was 5′-CCATGGCTCGAGCCCGGGTTACTTTACAACCCGTGG-3′. *Bam*H I and *Sma* I restriction sites are underlined. The PCR products were ligated to pLV-puro vector (a generous gift from Han's Lab at Xiamen University) by using the Gibson Assembly Kit (New England BioLabs, Ipswich, MA, USA) according to manufacturer's instructions. All constructs were verified by DNA-sequencing (Sangon Biotech, Shanghai, China).

### Preparation of monoclonal antibodies to ORF53

Hybridomas secreting anti-ORF53 mAbs were generated according to a standard protocol. Briefly, recombinant ORF53 was expressed in *E. coli* ER2566 strain by IPTG induction and purified by $Ni^{+}$ column chromatography. A group of five female BALB/c mice (Shanghai SLAC Laboratory Animal Co., Ltd., Shanghai, China) were immunized with recombinant ORF53 protein (100 μg per mouse) emulsified in Freund's adjuvant (Sigma, St. Louis, MO, USA) three times at two-week intervals. Then 50 μg of mixed ORF53 protein in phosphate buffered saline (PBS) was injected directly into the spleen of mice with the highest serum antibody titers against ORF53 three days prior to cell fusion. Spleen cell were separated from the immunized mice and fused with Sp2/0 myeloma cells using polyethylene glycol 1450 (Sigma). Hybridomas were cultured and selected in RPMI-1640 medium plus hypoxanthine-aminopterin-thymidine (HAT; Sigma) and 10% FBS. After 14 days, the supernatants were harvested and screened by Enzyme-linked immunosorbent assay (ELISA) against ORF53. The stable hybridomas secreting anti-ORF53 mAbs were injected into the abdominal cavity of mice. Finally, mAbs in the ascitic fluid were concentrated and purified by Protein A-Sepharose affinity chromatography.

### Immunoprecipitation and western blotting

293T cells were transfected with pLV-ORF7, pLV-ORF53 or pLV-puro vector alone, or co-transfected with pLV-ORF7 and pLV-ORF53, or pLV-ORF7 and pLV-GFP (a kind gift from Han's Lab), or pLV-ORF53 and pLV-GFP, respectively, using Lipofectamine® 2000 Transfection Reagent (Invitrogen, Carlsbad, CA, USA); while ARPE-19 cells were infected with cell-associated VZV WT at a multiplicity of infection (MOI) of 1. Both transfected 293T cells and infected ARPE-19 cells were collected after 2 days, and cell lysates were prepared in PBS containing 1% Triton X-100 and protease inhibitor mixture (Amresco, Solon, OH, USA). Immunoprecipitation of ORF7 or ORF53 protein were performed by incubating cell lysate supernatants with anti-ORF7 mAb 8H3 [described in our publication (Selariu et al., 2012; Jiang et al., 2017)] or anti-ORF53 mAb 2G12 and Dynabeads™ Protein G beads (Invitrogen) according to the manufacturer's instructions.

Samples of either cell lysates or beads in PBS were mixed with 6× protein loading buffer without reductant, boiled for 10 min, and then subjected to western blotting following a standard protocol. Briefly, samples were resolved by SDS-PAGE and electro-transferred from gels onto nitrocellulose membranes (Whatman, Florham Park, NJ, USA). After blocking in 5% skim milk in PBS for 1 h at room temperature, membranes were incubate at 37 °C for 1 h with primary antibodies of mouse anti-ORF7 mAb 8H3, mouse anti-ORF23 mAb 9A1 (unpublished data), mouse anti-ORF53 mAb 2G12, mouse anti-ORF66 mAb 1A12 (unpublished data) or mouse anti-GAPDH mAb 1E6D9 (Proteintech, Chicago, IL, USA), followed by incubation with an anti-mouse goat antiserum conjugated to horseradish peroxidase (HRP) (Santa Cruz Biotechnology, Santa Cruz, CA, USA) for another 30 min. Finally, protein bands were visualized by chemiluminescence (SuperSignal West Pico Chemiluminescent Substrate Kit; Pierce, Rockford, IL, USA).

### Immunofluorescence and confocal microscopy

ARPE-19 cells were seeded in 24-well tissue culture plates preplaced with one circular cover glass per well and then transfected with pLV-ORF7 or pLV-ORF53 alone, or co-transfected with pLV-ORF7 and pLV-ORF53, or infected with WT, 7D or 7R at an MOI of 0.01. After incubation at 37 °C in 5% $CO_2$ for 3 days, cells were fixed with paraformaldehyde for 30 min, permeabilized with 0.3% Triton X-100 in PBS for 10 min and blocked for 1 h with goat serum. Cells were then incubated with mouse anti-ORF7 mAb 8H3 or mouse anti-ORF53 mAb 2G12, together with a rabbit polyclonal antibody against trans-Golgi network (TGN) (TGN46; Novus Biologicals, Littleton, CO, USA) or a rabbit polyclonal antibody against ORF7 (7M; unpublished data) for 1 h, followed by incubation with both goat-anti-mouse-fluorescein isothiocyanate (FITC)- and/or goat-anti-rabbit-tetramethylrhodamine B isothiocyanate (TRITC)-labeled secondary antibodies (Sigma) for another 30 min. Cell nuclei were counterstained with 4', 6-diamidino-2-phenylindole, dihydrochloride (DAPI; Invitrogen), and cells on the cover glasses were observed using a Zeiss LSM 780 confocal microscope (Zeiss, Jena, Germany).

### Statistical analysis

Co-localizations of ORF7 and ORF53, ORF7 and TGN46, and ORF53 and TGN46 were quantified as Pearson's correlation coefficients using Image Pro Plus 6.0 software (Media Cybernetics, Inc., Silver Spring, MD, USA), and results were presented as mean ± standard error mean (SEM). Statistical analysis were performed with GraphPad Prism 5 (GraphPad Software Inc., La Jolla, CA, USA) using unpaired two-tailed Student's *t*-test.

## RESULTS

### Identification and characterization of VZV ORF53

To identify and characterize ORF53, we firstly generated anti-ORF53 mouse monoclonal antibodies (mAbs) using recombinant ORF53 as an antigen. For this purpose, the prokaryotic expression plasmid pTO-T7-ORF53 was constructed, and recombinant ORF53 was expressed in *E. coli* with IPTG induction and purified by $Ni^+$ column chromatography (Figure 1A). Preparation and characterization of mAbs against ORF53 was carried out through ELISA (data not shown), western blotting and indirect immunofluorescent microscopy as described in Materials and methods. One of anti-ORF53 mouse mAbs, clone 2G12, gave results with high sensitivity and low background. As shown in Figure 1B and 1C, mAb 2G12 reacted strongly with a protein with an apparent molecular of ~40 kDa in both VZV-infected ARPE-19 and pLV-ORF53-transfected 293T cell lysates, and no distinct

Figure 1. Characterization of the mAb 2G12 against VZV ORF53. (A) Recombinant ORF53 protein was expressed in *E. coli* by IPTG induction and purified by $Ni^{+}$ column chromatography (lane 2). The PageRuler™ Prestained Protein Ladder (Thermo Fisher Scientific) was used as marker (lane 1). Proteins were separated by SDS–PAGE and stained with Coomassie brilliant blue. (B) Evaluation of reactivity and specificity of mAb 2G12 against ORF53 by western blotting against extracts from uninfected (UI) and VZV-infected (WT) ARPE-19 cells (at day 2 post-infection). (C) Evaluation of reactivity and specificity of mAb 2G12 against ORF53 by western blotting against extracts from mock-, pLV-puro- and pLV-ORF53-transfected 293T cells (at day 2 post-transfection). (D) Expression of ORF53 detected by indirect immunofluorescence microscopy using mAb 2G12 in VZV-infected and pLV-ORF53-transfected ARPE-19 cells. ORF53 was detected in the juxtanuclear region (white arrow heads) of VZV-infected ARPE-19 cells but was dispersed throughout the cytoplasm of pLV-ORF53-transfected ones. Scale bar, 20 μm. In (A)–(C), the positions of molecular mass markers are shown to the left.

Figure 2. Interaction between ORF7 and ORF53 in infected and transfected cells. (A) Co-immunoprecipitation analysis of the interaction between ORF7 and ORF53 in VZV-infected cells. ARPE-19 cells were uninfected or infected with WT or 7D at MOI of 1. After 2 days, cells were lysed and ORF7 in the lysates was precipitated with anti-ORF7 mAb 8H3, followed by western blotting to detect ORF7 and ORF53. (B) Co-immunoprecipitation analysis of the interaction between ORF7 and ORF53 in transfected cells. 293T cells were untransfected, or transfected with pLV-ORF7 or pLV-ORF53 alone, or co-transfected with pLV-ORF7 and pLV-ORF53. Co-immunoprecipitation were performed with anti-ORF7 mAb 8H3 at 2 days post-transfection, followed by western blotting detection of ORF7 and ORF53. (C) ORF7 was detected in oligomeric forms with molecular weights approximating monomers, dimers and tetramers in VZV-infected ARPE-19 cells. The positions of molecular mass markers are shown to the left. WB, western blot; TCL, total cell lysate; IP, immunoprecipitation; M, protein marker (Thermo Fisher Scientific).

Figure 3. ORF7 and ORF53 co-localize in the trans-Golgi network of VZV-infected cells. ARPE-19 cells were infected with WT at MOI of 0.01 and fixed after 3 days. (A, B) Representative images of VZV-infected cells co-immunostained for ORF7 or ORF53 (TRITC/red) and TGN46 (FITC/green) show that both ORF7 and ORF53 co-localize with the TGN (yellow, marked by white arrow heads) in the context of infection. (C) Representative dual immunostaining for ORF53 (TRITC/red) and ORF7 (FITC/green) shows co-localization of ORF7 with ORF53 (yellow, marked by white arrow heads) in VZV-infected cells. Nuclei were stained with DAPI (blue). Scale bars, 20 μm.

protein band was observed in cell lysates from the control groups. Next, we examined the intracellular distribution of ORF53 by indirect immunofluorescence staining using mAb 2G12. The detected ORF53 showed juxtanuclear Golgi-like localization in VZV-infected ARPE-19 cells (Figure 1D, upper panels) but was dispersed in the cytoplasm of the plasmid-transfected ones (Figure 1D, lower panels); no staining was observed in negative control cells (data not shown). These results indicate that mAb 2G12 specifically detected ORF53, and thus we used this mAb for further experiments to study relationship between ORF7 and ORF53. Interestingly, ORF53 showed different intracellular localization in transfected and infected cells, thus indicating that there may be other viral protein(s) that influence its cellular distribution.

## ORF7 interacts with ORF53 in infected and transfected cells

To investigate the interaction between ORF7 and ORF53, we performed co-immunoprecipitation experiments in VZV-infected ARPE-19 cells and plasmid-transfected 293T cells, respectively. We tried to demonstrate interactions between ORF7 and ORF53 in both directions; as shown in Figure 2A and 2B, anti-ORF7 mAb 8H3 was capable of co-immunoprecipitating the ORF53 protein present in both the infected and transfected cell extracts. However, anti-ORF53 mAb 2G12 failed to co-precipitate ORF7 (data not shown), indicating possible overlap in the binding sites on ORF53 for this antibody and for ORF7. Additionally, no interaction between ORF7 or ORF53 with the control green fluorescent protein (GFP) in transfected cells or with another viral protein ORF66 in infected cells was observed (data not shown). Thus, these results indicate that ORF7 and ORF53 are probably present in a complex either in the absence or presence of other viral proteins. Interestingly, when immunoprecipitating ORF7 from VZV-infected cell lysates, we observed that ORF7 was not only present as a monomer of ~35 kDa,

Figure 4. ORF7 deletion does not affect expression of ORF53 in VZV-infected cells. ARPE-19 cells were infected with 7D, WT or 7R at MOI of 0.1 and lysed when cytopathic effect (CPE) reached ~50%. VZV proteins of ORF7, ORF23 and ORF53 as well as host GAPDH in the cell lysates were subsequently detected by western blotting.

but that it can also be detected in oligomeric forms with molecular weights approximating dimers and tetramers (Figure 2C). This finding may provide new evidence for ORF7 self-interaction, in addition to that suggested by previous yeast-two-hybrid studies (Uetz et al., 2006; Stellberger et al., 2010).

## ORF7 co-localizes with ORF53 in the TGN of infected cells

Subsequently, we investigated co-localization of ORF7 with ORF53 in VZV-infected ARPE-19 cells. Firstly, we found that both ORF7 and ORF53 co-localized well with the TGN46 protein (Figure 3A and 3B), which is widely used as a TGN marker in numerous studies (Barr et al., 2010; Donsante et al., 2011; Zhao et al., 2012; Charles et al., 2014; Nonnenmacher et al., 2015; Pillay et al., 2016). The Pearson's coefficients were 0.70 ± 0.09 and 0.68 ± 0.08 in the co-localized volume of TGN46 with ORF7 and ORF53, respectively (n = 10 in each group). These results indicate that ORF7 and ORF53 co-localized predominantly to the TGN of VZV-infected cells. To further confirm the co-localization of ORF7 and ORF53 in VZV-infected cells, we generated a rabbit polyclonal antibody against ORF7, and together with mouse anti-ORF53 mAb 2G12, we confirmed the co-localization of ORF7 with ORF53 in the infected ARPE-19 cells (Figure 3C)

Figure 5. ORF7 deletion affects the trans-Golgi network localization of ORF53 in VZV-infected cells. ARPE-19 cells were infected with 7D, WT or 7R at MOI of 0.01 and fixed after 3 days. Cells were subsequently co-immunostained for ORF53 (TRITC/red) and TGN46 (FITC/green), and nuclei were stained with DAPI (blue). Representative images show that ORF53 co-localized with the TGN (yellow, marked by white arrow heads) in cells infected with WT (middle panels) and 7R (lower panels). In contrast, ORF53 showed dispersed distribution in the cytoplasm of 7D-infected cells (upper panels), suggesting that ORF7 has a role in the TGN localization of ORF53. Scale bar, 20 μm.

Figure 6. Pearson's correlation coefficient values for co-localization of ORF53 and TGN46 in ARPE-19 cells infected with 7D, WT and 7R. The average Pearson's correlation coefficients ± SEM were calculated from ten randomly selected infected cells in each group. ****, $P < 0.0001$; ns, not significant.

with a Pearson's coefficient of 0.66 ± 0.10 (n = 10) by double-label staining and confocal microscopy analysis.

## TGN localization of ORF53 depends on ORF7 presence in infected cells

Based on above observations of the interaction and co-localization of ORF7 with ORF53, we speculated that ORF7 may be associated with the TGN localization of ORF53. Here we generated an ORF7-deleted VZV mutant, 7D, to test this hypothesis in the infected ARPE-19 cells. Firstly, by western blotting, we confirmed that ORF7 deletion didn't affect the expression of ORF53 (Figure 4). Then, by double-labeling immunofluorescent confocal microscopy, we found that, in comparison to TGN localization of ORF53 in cells infected with wild-type (WT) and revertant virus (7R) (Figure 5, middle and lower panels), ORF53 was dispersed throughout the cytoplasm of cells infected with 7D (Figure 5, upper panels) which was similar to that in transfected cells expressing ORF53 alone (Figure 1D, lower panels). The Pearson's coefficients for co-localization of ORF53 with TGN46 were calculated for WT, 7D and 7R, respectively (n = 10 in each group). As shown in Figure 6, Pearson's analysis revealed a statistically significant drop in the correlation coefficient in the absence of ORF7, indicating that ORF7 plays a role in the TGN localization of ORF53. On the other hand, in transfected ARPE-19 cells expressing ORF7, either alone (Figure 7A) or together with ORF53 (Figure 7B), ORF7 also displayed dispersing rather than juxtanuclear TGN localization, therefore there may be other viral proteins involved in mediating their normal cellular distribution during infection.

## DISCUSSION

Both ORF7 and ORF53 are conserved throughout the alphaherpesviruses. ORF7 is non-essential for VZV growth *in vitro* but has dual roles as virulence determinants in human skin and DRG tissue *ex vivo*/*in vivo* (Zhang et al., 2010; Selariu et al., 2012). Recently, several further studies have demonstrated that deletion of ORF7 does not prevent viral entry, viral replication and viral protein expression as well as retrograde transport of virus particles from axon terminals to somata but substantially affects secondary envelopment and cell-to-cell spread of VZV in differentiated neuronal cells *in vitro* (Grigoryan et al., 2012; Jiang et al., 2017). On the other hand, ORF53 is essential for VZV replication (Zhang et al., 2010), however, its characteristics have remain unstudied to date.

Previous yeast two-hybrid studies indicated that ORF7 and ORF53 may interact (Uetz et al., 2006; Stellberger et al., 2010). Here we aimed to investigate relationships between ORF7 and ORF53 in the context of VZV infection. Firstly, we prepared monoclonal antibodies against ORF53 and characterized it as a ~40 kDa viral protein which localizes in the juxtanuclear region of VZV-infected cells. Then, we confirmed the interaction between ORF7 and ORF53 by co-immunoprecipitation in both transfected and VZV-infected cells. We next performed immunostaining for ORF7 and/or ORF53 along with a TGN marker TGN46 and confirmed their co-localization predominantly in the TGN of VZV-infected cells. Moreover, interestingly, we found that ORF53 lost its TGN localization and became dispersed in the cytoplasm of cells infected with an ORF7-deleted VZV mutant. Therefore, ORF7 seems to play a role in recruiting ORF53 to the TGN during infection. However, both ORF7 and ORF53 demonstrated a dispersed distribution in the cytoplasm rather than specific TGN localization when expressing alone or together in transfected cells. Although we didn't perform three-color immunofluorescence staining to include the TGN marker in the co-transfection of ORF7 and ORF53 in this study (Figure 7B), we believe that the apparent difference in the cellular distribution of ORF7 and ORF53 in plasmid-transfected cells and VZV-infected cells is sufficient to differentiate. Thus these results indicated that there may be other viral proteins that are involved in the regulation of their cellular localization during VZV infection.

In conclusion, in this study, we confirmed the interaction of ORF7 with ORF53 by co-immunoprecipitation and co-localization by confocal microscopy, and we found that ORF7 is indispensable for the TGN localization of ORF53 in VZV-infected cells. Recently, the HSV-1 homologues of ORF7 and ORF53, UL51 and UL7 respectively, have been reported to interact forming a functional complex, which is important for efficient virus assembly and plaque formation as well as for stabilization of focal adhesions and maintaining host cell morphology

Figure 7. Localization of ORF7 and ORF53 complexes in transfected cells. (A) ARPE-19 cells were transfected with pLV-ORF53 or pLV-ORF7 alone and fixed 2 days following transfection, then cells were co-immunostained for ORF53 or ORF7 (TRITC/red) and TGN46 (FITC/green). (B) ARPE-19 cells were transfected with pLV-ORF7 alone, or co-transfected with pLV-ORF7 and pLV-ORF53. At 2 days post-transfection, cells were fixed and processed for immunostaining of ORF53 (TRITC/red) and ORF7 (FITC/green). Nuclei were stained with DAPI (blue). Representative images demonstrates that both ORF7 and ORF53 showed dispersed cytoplasmic distribution rather than TGN localization when expressing alone or together in transfected cells. Scar bar, 20 μm.

(Roller and Fetters, 2015; Albecka et al., 2017). Based on these previous data coupled with our present observation, it is tempting to speculate that ORF7 and ORF53 also function together and have a role in VZV infection, replication and disease pathogenesis, and thus future studies will be necessary to further characterize the functional significance of ORF7-ORF53 interaction as well as their localization in the TGN of infected cells.

## ACKNOWLEDGMENTS

This work was supported by the National Natural Science Foundation of China (No. 81601762), the National Science and Technology Major Project of Infectious Diseases (No. 2017ZX10304402), the National Science and Technology Major Projects for Major New Drugs Innovation and Development (No. 2017ZX09101005-005-003)

and the Scientific Research Foundation of State Key Laboratory of Molecular Vaccinology and Molecular Diagnostics (No. 2016ZY005).

## COMPLIANCE WITH ETHIC GUIDELINES

The authors declare they have no conflict of interest. This article does not contain any studies with human or animal subjects performed by any of the authors.

## AUTHOR CONTRIBUTIONS

WW, NSX, HZ and TC designed the experiments. WW, WKF, DQP, LLC, JHY, JL, CL and YQQ carried out the experiments. WW, WKF, HZ and TC analyzed the data and wrote the paper. All authors read and approved the final manuscript.

## OPEN ACCESS

This article is distributed under the terms of the Creative Commons Attribution 4.0 International License (https://creativecommons.org/licenses/by/4.0/), which permits unrestricted use, distribution, and reproduction in any medium, provided you give appropriate credit to the original author(s) and the source, provide a link to the Creative Commons license, and indicate if changes were made.
